# Predictors of intradialytic hypotension in critically ill patients undergoing kidney replacement therapy: a systematic review

**DOI:** 10.1186/s40635-024-00695-8

**Published:** 2024-11-21

**Authors:** Rafaella Maria C. Lyrio, Etienne Macedo, Raghavan Murugan, Arnaldo A. da Silva, Tess M. Calcagno, Estevão F. Sampaio, Rafael H. Sassi, Rogério da Hora Passos

**Affiliations:** 1 Universidade Salvador, Salvador, Brazil; 2 Division of Nephrology, Department of Medicine, University of California San Diego, San Diego, CA USA; 3 The Program for Critical Care Nephrology, Department of Critical Care Medicine, University of Pittsburgh School of Medicine, Pittsburgh, PA USA; 4 The Center for Research, Investigation, and Systems Modeling of Acute Illness (CRISMA), Department of Critical Care Medicine, University of Pittsburgh School of Medicine, Pittsburgh, PA USA; 5 Department of Critical Care, Hospital Israelita Albert Einstein, São Paulo, Brazil; 6 Department of Internal Medicine, Cleveland Clinic Foundation, Cleveland, OH USA; 7 Department of General Surgery, Hospital Geral Ernesto Simões Filho, Salvador, Brazil; 8 Department of Hematology, Hospital de Clínicas de Porto Alegre, Porto Alegre, Brazil; 9 Da Vita Tratamento Renal, São Paulo, Brazil

**Keywords:** Kidney replacement therapy, Hypotension, Acute kidney injury, Dialysis, Critical illness

## Abstract

**Background:**

This systematic review aims to identify predictors of intradialytic hypotension (IDH) in critically ill patients undergoing kidney replacement therapy (KRT) for acute kidney injury (AKI).

**Methods:**

A comprehensive search of PubMed was conducted from 2002 to April 2024. Studies included critically ill adults undergoing KRT for AKI, excluding pediatric patients, non-critically ill individuals, those with chronic kidney disease, and those not undergoing KRT. The primary outcome was identifying predictive tools for hypotensive episodes during KRT sessions.

**Results:**

The review analyzed data from 8 studies involving 2873 patients. Various machine learning models were assessed for their predictive accuracy. The Extreme Gradient Boosting Machine (XGB) model was the top performer with an area under the receiver operating characteristic curve (AUROC) of 0.828 (95% CI 0.796–0.861), closely followed by the deep neural network (DNN) with an AUROC of 0.822 (95% CI 0.789–0.856). All machine learning models outperformed other predictors. The SOCRATE score, which includes cardiovascular SOFA score, index capillary refill, and lactate level, had an AUROC of 0.79 (95% CI 0.69–0.89, *p* < 0.0001). Peripheral perfusion index (PPI) and heart rate variability (HRV) showed AUROCs of 0.721 (95% CI 0.547–0.857) and 0.761 (95% CI 0.59–0.887), respectively. Pulmonary vascular permeability index (PVPI) and mechanical ventilation also demonstrated significant diagnostic performance. A PVPI ≥ 1.6 at the onset of intermittent hemodialysis (IHD) sessions predicted IDH associated with preload dependence with a sensitivity of 91% (95% CI 59–100%) and specificity of 53% (95% CI 42–63%).

**Conclusion:**

This systematic review shows how combining predictive models with clinical indicators can forecast IDH in critically ill AKI patients undergoing KRT, with validation in diverse settings needed to improve accuracy and patient care strategies.

**Supplementary Information:**

The online version contains supplementary material available at 10.1186/s40635-024-00695-8.

## Background

Several clinical studies have documented a correlation between reduced arterial blood pressure and higher mortality rates among critically ill patients [1, 2]. Blood pressure (BP) is crucial for organ perfusion, enabling autoregulation and appropriate blood flow distribution. Hypotension, common in all kidney replacement therapy (KRT) modalities used in the intensive care unit (ICU), contributes to higher mortality rates during hospitalization and hampers kidney recovery [3, 4].

Hemodynamic instability in KRT patients is complex, beyond just fluid removal. Minimizing fluid shifts and using appropriate ultrafiltration rates in high-risk patients lack strong evidence, especially in continuous kidney replacement therapy (CKRT) [5, 6].

Risk factors for intradialytic hypotension (IDH) include a cardiovascular Sequential Organ Failure Assessment (SOFA) score of ≥ 1, the presence of two tissue hypoperfusion markers, a capillary refill time (CRT) of ≥ 3 s, and lactate levels exceeding 2 mmol/L [7]. The challenge in predicting IDH arises from the high prevalence of these risk factors among critically ill patients undergoing KRT, which complicates proactive management strategies. Additionally, these patients frequently experience distributive shock and reduced cardiac output, necessitating the use of vasopressors. These conditions, along with other comorbidities, further impair BP control mechanisms, making effective management of IDH particularly complex [5].

Even short periods of low BP can be harmful, suggesting a need to shift from reactive to proactive hemodynamic management. Current interventions to predict IDH are primarily based on observational studies, with limited guidance from randomized controlled trials (RCTs). Evaluating the effectiveness of these interventions on critical outcomes such as mortality, ICU and hospital length of stay, and renal recovery is paramount [8].

While awareness of the impact of hemodynamically significant IDH on outcomes in critically ill patients with acute kidney injury (AKI) undergoing KRT is growing, a significant gap persists in evidence-based strategies to optimize management.

This study aims to systematically review current predictive tools for hypotension in AKI patients undergoing KRT.

## Methods

The review protocol was registered on PROSPERO (Registration No: CRD42024509388) on 26 June 2024. The reporting adhered to the PRISMA guidelines [9], with the PRISMA checklist provided as Additional file 1: File A.

Our objective was to identify and examine predictors of IDH in critically ill adults undergoing KRT for AKI.

### Criteria for considering studies

#### Types of studies

We included observational studies and randomized controlled trials. Editorials and reviews were excluded.

#### Participant criteria

We focused on adult patients (> = 18 years of age) in a critical care setting undergoing KRT for AKI without chronic kidney disease.

#### Intervention types

Our primary goal was to evaluate the effectiveness of tools in predicting IDH in critically ill patients undergoing KRT for AKI.

#### Outcome measures

We categorized outcome measures into two primary types: statistical metrics and clinical outcomes.

Statistical metrics: area under the receiver operating characteristic curve (AUROC), sensitivity, specificity, positive predictive value (PPV), negative predictive value (NPV), kappa statistics, generalized estimating equations, linear regression and Chi-square analysis.

Clinical outcomes: Directly related to IDH, including its incidence, severity, and duration. Additional patient outcomes such as recovery times, mortality rates, and length of ICU stay.

### Search methods for identification of studies

Three independent reviewers (RL, ES, and TC) conducted a comprehensive electronic search in PubMed from 11th April 2002 to 30th April 2024. The search terms included “hemodialysis”, “hypotension”, “predictors”, “preload dependence”, “central venous pressure”, “ultrasonographic measurement”, “blood volume monitoring”, and “renal replacement therapy”. We limited the language to English.

### Data collection and analysis

#### Study selection

**Fig. 1:**
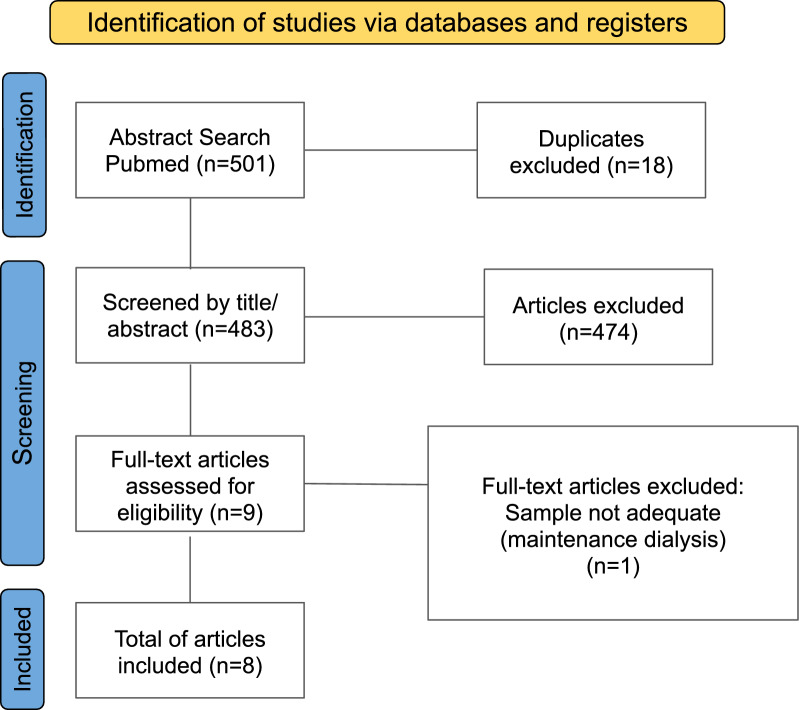
PRISMA flow diagram of studies assessed in the systematic review

Three independent reviewers (RL, TC, and ES) scrutinized the electronic search outcomes. Non-relevant studies were excluded in stages, as outlined in Fig. 1. Initial exclusions were based on titles and abstracts, followed by full-text reviews. Discrepancies were resolved collaboratively.

#### Data extraction and management

Data on study design, setting, population, intervention, and outcomes were independently extracted by three reviewers (RL, TC, and ES) using a pre-established form. Discrepancies were resolved through discussion, with a fourth reviewer (RS) available for arbitration if necessary.

#### Assessment of risk of bias in included studies

The quality of studies included in the systematic review was assessed by two researchers (AS, RP) using the Newcastle–Ottawa Scale (NOS), which scores studies from 0 to 9 points. The NOS evaluates the risk of bias in observational studies across three domains: (i) selection bias, (ii) comparability, and (iii) outcome assessment. For the randomized controlled trial, the risk of bias was analyzed using RevMan from Cochrane. This tool assesses bias in several areas: (i) selection bias, (ii) performance bias, (iii) detection bias, (iv) attrition bias, and (v) reporting bias. The detailed assessment of the risk of bias in included studies is available in Additional file 1: File B.

#### Data synthesis

Findings from the included studies were summarized descriptively, comparing the predictive accuracy of different tools and clinical predictors. Common themes and patterns in the data were identified, and the strength and consistency of the evidence were discussed.

## Results

An initial PubMed search yielded 501 articles, with 18 duplicates removed. Of the 474 papers excluded based on title and abstract, 9 articles underwent full-text review, with 1 excluded, resulting in 8 included articles. These comprised one prospective randomized controlled trial, six prospective observational studies, and one retrospective observational study, involving 2,873 patients. Study characteristics and results are detailed in Table 1.

**Table 1:** Characteristics and results of included studies

Author (year)	*N*, study design	Dialysis method	Definition of IDH hypotension	Predictors	Results
Kang et al. (2021)	2.349, retrospective observational study	CKRT	Reduction in MAP ≥ 20 mmHg and ≥ 30 mmHg within 6 h	Machine learning models: SVM, DNN, LGBM and XGB Scores: SOFA, APACHE II, MOSAIC	AUROC for MAP Δ20 and MAP Δ30, respectively: SVM: 0.807 (0.772–0.842); 0.830 (0.784–0.876) DNN: 0.822 (0.789–0.856); 0.835 (0.789–0.881) LGBM: 0.813 (0.780–0.847); 0.845 (0.802–0.888) XGB: 0.828 (0.796–0.861); 0.861 (0.822–0.900) SOFA: 0.500 (0.453–0.547); 0.496 (0.435–0.557) APACHE II: 0.546 (0.499–0.593); 0.592 (0.535–0.649) MOSAIC: 0.568 (0.522–0.615); 0.578 (0.518–0.638)
Passos et al. (2021)	248, prospective observational study	IHD	First occurrence of MAP < 65 mmHg during the session	Nephrologists' and intensivists’ perception Vascular pedicle width Cardiothoracic ratio Peripheral edema	Sensitivity: 21.5% and 22.8%; specificity: 45.6% and 47.3%; PPV: 15.6% and 16.8%; NPV: 55.4% and 56.7%; Agreement between (Kappa): 0.561 Sensitivity: 86.1% (95% CI 70.3–92.3); specificity: 55% (95% CI 43.2–73.2); PPV: 47.2% (95% CI 33.2–59.3); NPV: 89.4% (95% CI 74.2–94.5) Sensitivity: 67.1%; specificity: 19.5%; PPV: 28%; NPV: 55.9% Sensitivity: 77.2%; specificity: 59.8%; PPV: 47.3%; NPV: 84.9%
Bigé et al. (2020)	88, prospective observational study	IHD	BP drop requiring therapeutic intervention (i.e., fluid resuscitation, introduction or increase in vasopressors, decrease or cessation of ultrafiltration)	SOCRATE score	Occurrence percentage per score received: 10% (95% Cl [3%, 30%]), 33% (95% CI [15%, 58%]), 55% (95% CI [35%, 73%]), and 80% (95% Cl [55%, 93%]) for 0, 1, 2 and 3 parameters, respectively AUC: 0.79 (0.69–0.89), * p* < 0.0001)
Mostafa et al. (2019)	36, prospective observational study	IHD	20% reduction of MAP from baseline which required either initiation or increased rate of norepinephrine infusion	Peripheral perfusion index Heart rate variability Pulmonary edema	AUROC (95% CI): 0.721 (0.547–0.857); sensitivity: 100%; specificity: 56%; PPV: 80%; NPV: 100% AUROC (95% CI): 0.761(0.59–0.887); sensitivity: 61%; specificity: 91%; PPV: 92%; NPV: 56% OR: 13.75 (95% CI 1.4–136)
Bitker et al. (2016)	47, prospective observational study	IHD	First occurrence of MAP < 65 mmHg during the session	Pulmonary vascular permeability index Mechanical ventilation Heart rate Dialysate sodium concentration SAPS II SOFA score MAP at IDH onset Inotrope at IDH onset Time between ICU admission and IHD session	AUC: 0.68 (95% CI 0.53–0.83); sensitivity: 91% (95% CI 59–100); specificity: 53% (95% CI 42–63%); PPV: 19%; NPV 98% AUC: 0.69 (95% CI 0.54–0.85); sensitivity: 64% (95% CI 32–88); specificity: 75% (95% CI 65–83%); PPV: 23%; NPV 95% AUC: 0.60 (95% CI 0.38–0.81); sensitivity: 36% (95% CI 11–69); specificity: 91% (95% CI 83–96%); PPV: 31%; NPV 93% AUC: 0.63 (95% CI 0.45–0.81); sensitivity: 82% (95% CI 48–98); specificity: 45% (95% CI 45–55%); PPV: 15%; NPV 96% AUC: 0.46 (95% CI 0.30–0.62); sensitivity: 91% (95% CI 59–100); specificity: 27% (95% CI 19–37%); PPV: 13%; NPV 96% OR 1.34 (95% CI 1.13–1.76, * p* < 0.01) OR 0.93 (95% CI 0.84–0.98, * p* = 0.03) No: OR 1 Yes: OR 0.13 (95% CI 0.00–0.76, * p* = 0.04) OR 0.96 (95% CI 0.91–0.99, * p* = 0.04)
Du Cheyron et al. (2013)	74, prospective randomized controlled trial	IHD	SBP < 90 mmHg or a reduction > 40 mmHg from the baseline justifying therapeutic intervention	Blood volume (BV) monitoring Blood temperature monitoring SOFA score Vasopressors at IHD onset SBP at IHD onset	Treatment B versus A: OR 0.98 (95% CI 0.63–1.97, * p* = 0.93); treatment C versus A: OR 0.84 ( 95% CI, 0.55–1.29, * p* = 0.48) OR 1.11 (95% CI 1.02–1.17, * p* = 0.01) OR 3.60 (95% CI 1.95–6.63, * p* < 0.0001) OR 0.98 (95% CI 0.96–0.99, * p* = 0.004)
Tanguay et al. (2007)	11, prospective observational study	IHD	SBP < 90 mmHg or a decrease of > = 30 mmHg and/or a MAP < 70 mmHg	BV monitoring	BV and MAP: correlation = 0.075, variance explained = 0.60%, * p* = 0.70 BV and SBP: correlation = 0.327, variance explained = 11%, * p* = 0.08 CBV and MAP: correlation = 0.144, variance explained = 2.10%, * p* = 0.083 CBV and SBP: correlation = 0.151, variance explained = 2.30%, * p* = 0.068 BV slope inter-rater agreement: IHD run one: *k* = 0.554, IHD run two: * k* = 0.033, IHD run three: * k* = 0.542, overall agreement = 51%
Tonelli et al. (2002)	20, prospective observational study	IHD	MAP < 70 mmHg for at least 5 min duration and SBP < 100 mmHg	BV monitoring	RBV and hypotension: kappa < 0.3

IDH, intradialytic hypotension; CKRT, continuous kidney replacement therapy; IHD, intermittent hemodialysis; MAP, mean arterial pressure; BP, blood pressure; SPB, systolic blood pressure; SVM, support vector machine; DNN, deep neural network; LGBM, light gradient boosting machine; XGB, extreme gradient boosting; SOFA, sequential organ failure assessment; APACHE II, Acute Physiology and Chronic Health Evaluation II; MOSAIC, Mortality Scoring System for Acute Kidney Injury with CKRT; SOCRATE, SOFA score, index CRT > 3 s, and lactate > 2 mmol/L; SAPS II, Simplified Acute Physiology Score II; ICU, intensive care unit; BV, blood volume; AUROC, area under the receiver operating characteristic curve; PPV, positive predictive value; NPV, negative predictive value; AUC, area under the curve; OR, odds ratio; CBV, critical blood volume; RBV, relative blood volume

### *Dialysis* modality and definition of intradialytic hypotension

Intermittent hemodialysis (IHD) was predominantly used [7, 10–15], with one study employing CKRT [16]. Definitions of IDH varied, with some using mean arterial pressure (MAP) thresholds (< 65 mmHg or < 70 mmHg) and others using systolic blood pressure (SBP) criteria (< 90 mmHg or < 100 mmHg) [10, 12–15]. The CKRT study defined IDH as MAP reductions of ≥ 20 mmHg or ≥ 30 mmHg within 6 h (16). Other definitions included sessional decreases of ≥ 30 mmHg or > 40 mmHg during IHD sessions and a 20% MAP reduction from baseline necessitating fluid resuscitation, vasopressor administration, or ultrafiltration adjustment [7, 11, 13, 14].

### Predictors of IDH and their performance

A total of 28 predictors were identified, most evaluated by individual studies. Notably, the SOFA score was assessed in three studies and demonstrated significance in predicting outcomes [12, 13, 16]. Each incremental point increase in the SOFA score on the day of IDH was associated with higher odds of the outcome, with odds ratios of 1.11 (95% CI 1.02–1.17, *p* = 0.01) and 1.34 (95% CI 1.13–1.76, *p* < 0.01) [12]. Additionally, the SOCRATE score, a composite measure incorporating cardiovascular SOFA score, index CRT > 3s, and lactate > 2mmol/L, exhibited predictive value, with an area under the curve (AUC) of 0.79 (95% CI 0.69–0.89, *p* < 0.0001) [7].

Machine learning models consistently outperformed traditional scores in recent research. Notably, Extreme Gradient Boosting (XGB) demonstrated the highest predictive performance, with an AUROC of 0.828 (95% CI 0.796–0.861) for MAP Δ20 and 0.861 (95% CI 0.822–0.900) for MAP Δ30. The Deep Neural Network (DNN) model achieved AUROCs of 0.822 (0.789–0.856) for MAP Δ20 and 0.835 (0.789–0.881) for MAP Δ30. In contrast, traditional scores such as SOFA, Acute Physiology and Chronic Health Evaluation II (APACHE II), and the mortality scoring system for acute kidney injury with CKRT (MOSAIC) demonstrated lower AUROC values [16]. For instance, the Simplified Acute Physiology Score II (SAPS II) exhibited poor discriminative ability with an AUROC of 0.46 (95% CI 0.30–0.62) [12].

Furthermore, recent studies have explored the correlation between blood volume (BV) monitoring and hemodynamic parameters, revealing weak and statistically insignificant correlations with MAP and SBP. Inter-rater agreement for BV slope varied across runs, with moderate consistency in some instances [13–15].

Among monitored parameters, heart rate variability (HRV), especially when ≤ 24, demonstrated discriminative ability with an AUROC of 0.761 (95% CI 0.59–0.887), while heart rate (HR) had poorer discriminative ability with an AUROC of 0.60 (95% CI 0.38–0.81) [11, 12]. Peripheral perfusion index (PPI) also showed promising predictive ability, with an AUROC of 0.721 (95% CI 0.547–0.857) [11]. Pulmonary vascular permeability index (PVPI) had an AUROC of 0.68 (95% CI 0.53–0.83), with a sensitivity of 91% (95% CI 59–100) and specificity of 53% (95% CI 42–63%) when PVPI ≥ 1.6 at the onset of IHD [12]. Monitoring of MAP or SBP at the onset of the IHD session showed lower levels in sessions with hypotensive episodes compared to those without [12, 13]. Dialysate sodium concentration exhibited an AUC of 0.63 (95% CI 0.45–0.81), indicating fair discriminative ability, with a sensitivity of 82% (95% CI 48–98) and a specificity of 45% (95% CI 45–55%). Its PPV was low at 15%, while the NPV was high at 96% [12]. ICU-related parameters were also explored. Mechanical ventilation was a significant predictor with an AUC of 0.69 (95% CI 0.54–0.85) [12]. The need for vasopressors at the onset of the IHD session, along with a shorter time between ICU admission and the IHD session, were identified as independent predictors of IDH [13]. The use of inotrope was not associated with an increased incidence of IDH, as indicated by an OR of 1 [12]. Vascular pedicle width (VPW) exhibited a sensitivity of 86.1% (95% CI 70.3–92.3), specificity of 55% (95% CI 43.2–73.2), with a ROC curve AUC of 0.81 (95% CI 0.75–0.84), *p* < 0.0001 to predict the absence of hypotension and best accuracy at a cut-off value of 70mm. The cardiothoracic ratio (CRT) showed lower sensitivity (67.1%) and specificity (19.5%) [10]. The presence of pulmonary edema was associated with higher odds of the outcome, with an odds ratio of 13.75 (95% CI 1.4–136) [11]. Peripheral edema had a sensitivity of 77.2% and a specificity of 59.8%. Nephrologists' and intensivists’ judgments of hypervolemia had sensitivities of 21.5% and 22.8%, respectively, with specificities of 45.6% and 47.3%, and weak agreement (kappa: 0.561) [10].

### Analysis of outcomes

The incidence of IDH ranged from 4 to 70% depending on the definition utilized in the study [7, 10–16]. One study found that hypotension, defined as a reduction in MAP ≥ 20 mmHg within 6 h, occurred in 29% of patients, and a reduction ≥ 30 mmHg was seen in 14%. Within 1 h, these rates were lower at 10% and 4%, respectively. Greater decreases in MAP correlated with higher ICU mortality risk, emphasizing the importance of maintaining adequate blood pressure during CKRT [16]. Recovery times and length of ICU stay were not captured in the present studies.

Hypotension varied significantly among IHD sessions—30%, 31.9%, 57%, and 70% based on MAP and 18% and 34% on SBP—with one report of higher rates in the second IHD run [10, 12, 14, 15]. Despite frequent hypotensive episodes, treatment was often not given, especially in the third IHD run [14]. Meanwhile, when IDH was defined as a BP drop requiring therapeutic intervention (i.e., fluid resuscitation, introduction or increase in vasopressors, decrease or cessation of ultrafiltration), occurrence across studies was 16.6%, 23%, and 64%, mainly during the initial hour and was more common in the first session than in later (43% vs 13%, *p* < 0.0001) [7, 11, 13].

None of the studies evaluated an intervention or a care protocol based on the results provided by the predictive tool for IDH. No randomized studies were found testing clinical interventions based on the predictive tool for hypotension.

## Discussion

This systematic review synthesizes current evidence on predictors of IDH in critically ill patients undergoing KRT for AKI. The findings emphasize the potential of machine learning models and various clinical parameters in predicting IDH, a significant complication associated with increased mortality and impaired kidney recovery.

### Machine learning models

The review highlights the superior performance of machine learning models in predicting IDH compared to traditional clinical scores. Notably, the XGB and DNN models achieved the highest predictive accuracies with AUROC values of 0.828 and 0.822, respectively. These models leverage complex interactions within data, enabling more nuanced predictions than linear models like SOFA or APACHE II [16]. Previous studies have also shown the efficacy of machine learning in medical prognostication. For example, Cheng et al. found that machine-learning algorithms significantly outperformed conventional methods in predicting mortality in ICU patients with sepsis [17]. Similarly, Tomasev et al. highlighted the potential of deep learning models to predict AKI up to 48 h in advance [18]. The ability of machine learning models to process large datasets and identify patterns not evident through traditional statistical methods suggests a paradigm shift in predicting hemodynamic instability during KRT.

### Clinical parameters and traditional scores

The SOCRATE score, which combines the cardiovascular SOFA score, CRT, and lactate levels, demonstrated moderate predictive accuracy with an AUROC of 0.79 among traditional clinical parameters [7]. This score effectively incorporates markers of tissue perfusion and organ failure, directly relevant to the hemodynamic challenges faced during KRT. While the AUROC values of 0.761 and 0.721 for HRV and PPI may not indicate strong predictive power, they still suggest a moderate level of utility in clinical practice, particularly when considered alongside other clinical parameters [11]. HRV measures the variability in time between heartbeats, reflecting autonomic nervous system function, while PPI assesses the strength of blood flow to peripheral tissues. Similar findings have been reported in the literature, where HRV and PPI have been used to assess hemodynamic status in various critical care settings [19, 20]. Integrating these parameters can enhance early detection of hemodynamic deterioration, guide therapeutic interventions, and ultimately improve patient outcomes.

Conversely, traditional scoring systems like SOFA and APACHE II showed lower predictive accuracies [16]. The poor performance of these scores may be attributed to their design for broader clinical prognostication rather than the specific context of IDH during KRT. This highlights the need for more specialized tools tailored to the unique pathophysiology of AKI and KRT. Recent literature has also questioned the specificity of traditional scoring systems in predicting outcomes in critically ill patients, emphasizing the need for context-specific predictive models [21, 22]. We acknowledge that while APACHE II was used for mortality risk assessment, it may underestimate mortality in today’s critically ill patients. Recent evidence suggests that models like SAPS III, which incorporate a wider range of clinical parameters, offer improved predictive accuracy [23].

### Blood volume monitoring and vascular parameters

The review also examined the role of BV monitoring and various vascular parameters. While BV monitoring showed weak correlations with IDH, other vascular indicators like the PVPI and VPW displayed moderate predictive capabilities [10, 12–15]. Specifically, a PVPI ≥ 1.6 at the onset of IDH was associated with high sensitivity (91%) for predicting IDH, though specificity was moderate (53%) [12]. These findings align with studies by Monnet et al. and Boyd et al., which have shown the utility of dynamic preload indices in predicting fluid responsiveness and hemodynamic instability in critically ill patients [24, 25].

Additionally, other studies have emphasized the importance of continuous monitoring of vascular parameters to tailor fluid management strategies and improve patient outcomes. The dynamic nature of indices like PVPI and VPW allows clinicians to assess real-time changes in patient status, providing a more responsive approach to treatment. Furthermore, incorporating these vascular parameters into routine monitoring could potentially reduce the incidence of IDH by enabling earlier interventions. This comprehensive approach underscores the need for advanced hemodynamic monitoring in managing critically ill patients undergoing dialysis. Moreover, research by Magder and Michard has further validated the predictive value of these vascular parameters in managing fluid therapy and preventing hemodynamic instability [26, 27].

### Net ultrafiltration rate

The rate of net ultrafiltration (UF) has been identified as a critical factor influencing the development of IDH. Studies have shown that higher UF rates are associated with an increased risk of IDH due to rapid volume depletion, leading to decreased venous return and cardiac output. In this review, the role of the UF rate as a predictor of IDH was not explicitly detailed, which suggests an area for further research. Adjusting UF rates based on individual patient hemodynamic status and tolerance could mitigate the risk of IDH. Recognizing UF tolerance, as proposed by Ramírez-Guerrero et al., emphasizes the need for careful monitoring and adjustment to balance fluid removal with hemodynamic stability [28].

### ICU-related parameters

ICU-related parameters such as mechanical ventilation and the need for vasopressors also emerged as significant predictors of IDH [12, 13]. These factors reflect the overall severity of illness and the hemodynamic instability common in critically ill patients. The inclusion of these parameters in predictive models could enhance their accuracy and clinical relevance. Previous studies have identified mechanical ventilation and vasopressor use as key indicators of poor prognosis in critically ill patients, further supporting their relevance in predicting IDH [29, 30].

Additionally, there is evidence suggesting that a shorter duration between ICU admission and the commencement of IHD may heighten the risk of IDH [12]. This could potentially be attributed to several factors and might also reflect a reduced period for comprehensive ICU care, potentially impacting patient management strategies and overall stability during dialysis sessions [29].

Moreover, the presence of pulmonary edema in critically ill patients can predict IDH [11]. Pulmonary edema indicates fluid overload and compromised cardiac function, which exacerbates hemodynamic instability during dialysis. Studies have demonstrated that patients with pulmonary edema are at higher risk for cardiovascular complications, including IDH, due to the additional strain on the cardiovascular system [29, 30].

### Insights and comprehensive analysis

The high incidence of IDH in critically ill patients with AKI undergoing KRT and its link to increased ICU mortality highlight a need for effective preventive interventions [16]. The current reliance on nephrologists' and intensivists' judgments for predicting IDH is unreliable, suggesting a shift toward evidence-based predictive tools [10].

This systematic review acknowledges the variability in methodologies across diverse studies exploring machine learning models and clinical parameters. While the use of aggregated data introduces complexities, it also enhances our understanding of IDH in critically ill patients, helping to identify consistent trends and gaps in the literature. By synthesizing these findings, we aim to stimulate discussions on improving predictive accuracy and encourage future research to refine methodologies and develop standardized approaches, ultimately advancing the management of hypotension in this vulnerable population.

While most research focuses on IHD, CKRT, which is more common in ICU settings, also faces hypotension challenges, particularly when transitioning between modalities [31].

Findings indicate that IDH is influenced by both dialysis settings and patient-specific factors [13, 15]. IDH often occurs within the first hour of IHD, even without significant fluid removal, suggesting that UF alone does not account for all cases [7, 12]. Therefore, multimodal strategies, such as early administration of vasopressors and careful fluid management, may be necessary [11].

Identifying patients sensitive to fluid removal could help tailor interventions, including adjusting UF rates or transitioning to CKRT [11, 12]. However, the reviewed studies did not assess the effectiveness of interventions based on predictive tools. Future RCTs are needed to determine whether these strategies can reduce IDH and improve outcomes [32–34].

### Limitations

We acknowledge the limitations of our review, particularly the heterogeneity in definitions of IDH and the inclusion of different renal replacement therapies (IHD and CKRT). This variability may affect the comparability of our findings. Additionally, our analysis lacks a training and validation dataset for predictive models. Future research should focus on standardized IDH definitions and structured datasets to enhance the robustness and applicability of predictive tools in clinical practice.

The analysis of IDH is constrained by the exclusion of several critical factors, including dialysate sodium concentration, dialysate temperature, preload measurements, and catecholamine dosing. Future research should incorporate these variables into predictive models to enhance both accuracy and clinical relevance. By expanding the range of factors considered, we can improve our understanding of the multifactorial nature of IDH and develop more effective management strategies for this significant complication in critically ill patients undergoing renal replacement therapy.

The exclusion of non-English language studies may have led to selection bias, potentially overlooking relevant research published in other languages. The review also did not explicitly address the impact of net UF rates on IDH, a critical factor influencing hemodynamic stability during KRT. Further investigation into the role of UF rates and the development of adaptive UF strategies tailored to individual patient needs is necessary. Lastly, the integration of machine learning models into clinical practice requires rigorous validation and careful consideration of ethical and practical issues, such as data privacy and the need for clinician training in using these advanced tools.

### Implications for clinical practice

While the integration of machine learning models and specific clinical parameters holds promise for enhancing the management of IDH in critically ill patients, it is imperative that further randomized controlled trials are conducted to validate their effectiveness and ensure safe implementation in clinical practice. A study by Johnson et al. demonstrated that implementing machine learning-based decision support systems in the ICU improved patient outcomes and workflow efficiency, underscoring the potential benefits of such technologies in clinical practice [35].

## Conclusion

This review highlights the potential of combining advanced predictive models with traditional clinical indicators to forecast intradialytic hypotension in critically ill patients undergoing renal replacement therapy for acute kidney injury. Further validation across diverse clinical settings is crucial to refine predictive accuracy and improve patient care strategies.

## Supplementary Information

Supplementary file1 (DOCX 162 KB)

## Data Availability

The dataset supporting the conclusions of this article is included within the article and its additional file 1.
