# Supplementary material for: Predictors of intradialytic hypotension in critically ill patients undergoing kidney replacement therapy: a systematic review
Source: Intensive Care Med Exp. 2024 Nov 21;12:106. doi: 10.1186/s40635-024-00695-8 (PMC11582124; doi:10.1186/s40635-024-00695-8)
Supplement: Supplementary file 1 — Supplementary file1 (DOCX 162 KB) [file 40635_2024_695_MOESM1_ESM.docx]

**Predictors of Intradialytic Hypotension in Critically Ill Patients Undergoing Kidney Replacement Therapy: A Systematic Review**

**Additional files**

Rafaella Maria C Lyrio^1^, Etienne Macedo^2^, Raghavan Murugan^3,4^, Arnaldo A da Silva^5^, Tess M Calcagno^6^, Estevão F Sampaio^7^, Rafael H Sassi^8^, Rogério H Passos^5,9*^

^1^Universidade Salvador, Salvador, Brazil

^2^Division of Nephrology, Department of Medicine, University of California San Diego, CA, USA

^3^The Program for Critical Care Nephrology, Department of Critical Care Medicine, University of Pittsburgh School of Medicine, Pittsburgh, PA, USA

^4^The Center for Research, Investigation, and Systems Modeling of Acute Illness (CRISMA), Department of Critical Care Medicine, University of Pittsburgh School of Medicine, Pittsburgh, PA, USA

^5^Department of Critical Care, Hospital Israelita Albert Einstein, São Paulo, Brazil

^6^Department of Internal Medicine, Cleveland Clinic Foundation, OH, USA

^7^Department of General Surgery, Hospital Geral Ernesto Simões Filho, Salvador, Brazil

^8^Department of Hematology, Hospital de Clínicas de Porto Alegre, Porto Alegre, Brazil

^9^Da Vita Tratamento Renal, São Paulo, Brazil

***Corresponding author:**

Rogério H Passos

[oiregorpassos@yahoo.com.br](mailto:oiregorpassos@yahoo.com.br)

**Additional File A**. PRISMA 2020 checklist.

**Additional File B.** Assessment of risk of bias in included studies.

**Additional File A.** PRISMA 2020 checklist.

| **Section and Topic** | **Item #** | **Checklist item** | **Location where item is reported** |
| --- | --- | --- | --- |
| **TITLE** | | |  |
| Title | 1 | Identify the report as a systematic review. | Title page |
| **ABSTRACT** | | |  |
| Abstract | 2 | See the PRISMA 2020 for Abstracts checklist. | p.1-2 |
| **INTRODUCTION** | | |  |
| Rationale | 3 | Describe the rationale for the review in the context of existing knowledge. | p. 2 |
| Objectives | 4 | Provide an explicit statement of the objective(s) or question(s) the review addresses. | p. 3 |
| **METHODS** | | |  |
| Eligibility criteria | 5 | Specify the inclusion and exclusion criteria for the review and how studies were grouped for the syntheses. | p. 3 |
| Information sources | 6 | Specify all databases, registers, websites, organisations, reference lists and other sources searched or consulted to identify studies. Specify the date when each source was last searched or consulted. | p. 4 |
| Search strategy | 7 | Present the full search strategies for all databases, registers and websites, including any filters and limits used. | p. 4 |
| Selection process | 8 | Specify the methods used to decide whether a study met the inclusion criteria of the review, including how many reviewers screened each record and each report retrieved, whether they worked independently, and if applicable, details of automation tools used in the process. | p. 4 |
| Data collection process | 9 | Specify the methods used to collect data from reports, including how many reviewers collected data from each report, whether they worked independently, any processes for obtaining or confirming data from study investigators, and if applicable, details of automation tools used in the process. | p. 3 |
| Data items | 10a | List and define all outcomes for which data were sought. Specify whether all results that were compatible with each outcome domain in each study were sought (e.g. for all measures, time points, analyses), and if not, the methods used to decide which results to collect. | p. 3-4 |
|  | 10b | List and define all other variables for which data were sought (e.g. participant and intervention characteristics, funding sources). Describe any assumptions made about any missing or unclear information. | p. 4 |
| Study risk of bias assessment | 11 | Specify the methods used to assess risk of bias in the included studies, including details of the tool(s) used, how many reviewers assessed each study and whether they worked independently, and if applicable, details of automation tools used in the process. | p. 4, Ad. File B |
| Effect measures | 12 | Specify for each outcome the effect measure(s) (e.g. risk ratio, mean difference) used in the synthesis or presentation of results. | p. 4 |
| Synthesis methods | 13a | Describe the processes used to decide which studies were eligible for each synthesis (e.g. tabulating the study intervention characteristics and comparing against the planned groups for each synthesis (item #5)). | p. 5 |
|  | 13b | Describe any methods required to prepare the data for presentation or synthesis, such as handling of missing summary statistics, or data conversions. | p. 5 |
|  | 13c | Describe any methods used to tabulate or visually display results of individual studies and syntheses. | p. 5 |
|  | 13d | Describe any methods used to synthesize results and provide a rationale for the choice(s). If meta-analysis was performed, describe the model(s), method(s) to identify the presence and extent of statistical heterogeneity, and software package(s) used. | p. 5 |
|  | 13e | Describe any methods used to explore possible causes of heterogeneity among study results (e.g. subgroup analysis, meta-regression). | p. 5 |
|  | 13f | Describe any sensitivity analyses conducted to assess robustness of the synthesized results. | p. 5 |
| Reporting bias assessment | 14 | Describe any methods used to assess risk of bias due to missing results in a synthesis (arising from reporting biases). | p. 4 |
| Certainty assessment | 15 | Describe any methods used to assess certainty (or confidence) in the body of evidence for an outcome. | p. 4 |
| **RESULTS** | | |  |
| Study selection | 16a | Describe the results of the search and selection process, from the number of records identified in the search to the number of studies included in the review, ideally using a flow diagram. | Fig. 1, p. 5 |
|  | 16b | Cite studies that might appear to meet the inclusion criteria, but which were excluded, and explain why they were excluded. | Fig. 1, p. 5 |
| Study characteristics | 17 | Cite each included study and present its characteristics. | Table 1 |
| Risk of bias in studies | 18 | Present assessments of risk of bias for each included study. | Ad. File B |
| Results of individual studies | 19 | For all outcomes, present, for each study: (a) summary statistics for each group (where appropriate) and (b) an effect estimate and its precision (e.g. confidence/credible interval), ideally using structured tables or plots. | Table 1 |
| Results of syntheses | 20a | For each synthesis, briefly summarise the characteristics and risk of bias among contributing studies. | pp. 5-7, Ad. File B |
|  | 20b | Present results of all statistical syntheses conducted. If meta-analysis was done, present for each the summary estimate and its precision (e.g. confidence/credible interval) and measures of statistical heterogeneity. If comparing groups, describe the direction of the effect. | pp. 5-7 |
|  | 20c | Present results of all investigations of possible causes of heterogeneity among study results. | pp. 5-7 |
|  | 20d | Present results of all sensitivity analyses conducted to assess the robustness of the synthesized results. | pp. 5-7 |
| Reporting biases | 21 | Present assessments of risk of bias due to missing results (arising from reporting biases) for each synthesis assessed. | Ad. File B |
| Certainty of evidence | 22 | Present assessments of certainty (or confidence) in the body of evidence for each outcome assessed. | pp. 5-7 |
| **DISCUSSION** | | |  |
| Discussion | 23a | Provide a general interpretation of the results in the context of other evidence. | pp. 7-10 |
|  | 23b | Discuss any limitations of the evidence included in the review. | p. 10-11 |
|  | 23c | Discuss any limitations of the review processes used. | p. 10-11 |
|  | 23d | Discuss implications of the results for practice, policy, and future research. | p. 11 |
| **OTHER INFORMATION** | | |  |
| Registration and protocol | 24a | Provide registration information for the review, including register name and registration number, or state that the review was not registered. | p. 3 |
|  | 24b | Indicate where the review protocol can be accessed, or state that a protocol was not prepared. | p. 3 |
|  | 24c | Describe and explain any amendments to information provided at registration or in the protocol. | p. 3 |
| Support | 25 | Describe sources of financial or non-financial support for the review, and the role of the funders or sponsors in the review. | p. 12 |
| Competing interests | 26 | Declare any competing interests of review authors. | p. 11 |
| Availability of data, code and other materials | 27 | Report which of the following are publicly available and where they can be found: template data collection forms; data extracted from included studies; data used for all analyses; analytic code; any other materials used in the review. | p. 11 |

*From:* Page MJ, McKenzie JE, Bossuyt PM, Boutron I, Hoffmann TC, Mulrow CD, et al. The PRISMA 2020 statement: an updated guideline for reporting systematic reviews. BMJ 2021;372:n71. doi: 10.1136/bmj.n71 For more information, visit: <http://www.prisma-statement.org/>

**Additional File B.** Assessment of risk of bias in included studies

**NEWCASTLE - OTTAWA QUALITY ASSESSMENT SCALE**

**CASE CONTROL STUDIES**

**Prediction of hemodynamic tolerance of intermittent hemodialysis in critically ill patients: a cohort study**

Selection

1. Representativeness of the exposed cohort

**- a) truly representative of the average critically ill patients undergoing renal replacement therapy in the community**

- b) somewhat representative of the average critically ill patients in the community

- c) selected group of users, e.g., nurses, volunteers

- d) no description of the derivation of the cohort

2. Selection of the non-exposed cohort

**- a) drawn from the same community as the exposed cohort**

- b) drawn from a different source

- c) no description of the derivation of the non-exposed cohort

3. Ascertainment of exposure

**- a) secure record (e.g., surgical records)**

- b) structured interview

- c) written self-report

- d) no description

4. Demonstration that outcome of interest was not present at start of study

**- a) yes**

- b) no

Comparability

1. Comparability of cohorts on the basis of the design or analysis

**- a) study controls for the most important factor (e.g., use of norepinephrine)**

**- b) study controls for any additional factor (e.g., presence of sepsis)**

Outcome

1. Assessment of outcome

**- a) independent blind assessment**

**- b) record linkage (e.g., hospital records)**

- c) self-report

- d) no description

2. Was follow-up long enough for outcomes to occur

**- a) yes**

- b) no

3. Adequacy of follow-up of cohorts

**- a) complete follow-up – all subjects accounted for**

- b) subjects lost to follow-up are not described

- c) different follow-up rates between groups and no description

Overview

This table summarizes the quality assessment of the study "Prediction of hemodynamic tolerance of intermittent haemodialysis in critically ill patients: a cohort study" using the Newcastle-Ottawa Scale. The study was well-conducted with adequate representativeness of the exposed and non-exposed cohorts, secure ascertainment of exposure and outcomes, and control for important factors such as the use of norepinephrine and presence of sepsis. The main limitation is the lack of detailed description of the derivation of the non-exposed cohort.

**Bedside prediction of intradialytic hemodynamic instability in critically ill patients: the SOCRATE study**

Selection

1. Representativeness of the exposed cohort

**- a) truly representative of the average critically ill patients undergoing renal replacement therapy in the** community

- b) somewhat representative of the average critically ill patients in the community

- c) selected group of users, e.g., nurses, volunteers

- d) no description of the derivation of the cohort

2. Selection of the non-exposed cohort

**- a) drawn from the same community as the exposed cohort**

- b) drawn from a different source

- c) no description of the derivation of the non-exposed cohort

3. Ascertainment of exposure

**- a) secure record (e.g., surgical records)**

- b) structured interview

- c) written self-report

- d) no description

4. Demonstration that outcome of interest was not present at start of study

**- a) yes**

- b) no

Comparability

1. Comparability of cohorts on the basis of the design or analysis

**- a) study controls for the most important factor (e.g., use of norepinephrine)**

**- b) study controls for any additional factor (e.g., presence of sepsis)**

Outcome

1. Assessment of outcome

**- a) independent blind assessment**

**- b) record linkage (e.g., hospital records)**

- c) self-report

- d) no description

2. Was follow-up long enough for outcomes to occur

**- a) yes**

- b) no

3. Adequacy of follow-up of cohorts

**- a) complete follow-up – all subjects accounted for**

- b) subjects lost to follow-up are not described

- c) different follow-up rates between groups and no description

Overview

This table summarizes the quality assessment of the study "Bedside prediction of intradialytic hemodynamic instability in critically ill patients: the SOCRATE study" using the Newcastle-Ottawa Scale.

**Evaluation of peripheral perfusion index and heart rate variability as early predictors for intradialytic hypotension in critically ill patients**

Selection

1. Representativeness of the exposed cohort

**- a) truly representative of the average critically ill patients undergoing renal replacement therapy in the community**

- b) somewhat representative of the average critically ill patients in the community

- c) selected group of users, e.g., nurses, volunteers

- d) no description of the derivation of the cohort

2. Selection of the non-exposed cohort

**- a) drawn from the same community as the exposed cohort**

- b) drawn from a different source

- c) no description of the derivation of the non-exposed cohort

3. Ascertainment of exposure

**- a) secure record (e.g., surgical records)**

- b) structured interview

- c) written self-report

- d) no description

4. Demonstration that outcome of interest was not present at start of study

**- a) yes**

- b) no

Comparability

1. Comparability of cohorts based on the design or analysis

**- a) study controls for the most important factor (e.g., presence of pulmonary oedema)**

**- b) study controls for any additional factor (e.g., use of norepinephrine)**

Outcome

1. Assessment of outcome

**- a) independent blind assessment**

- b) record linkage (e.g., hospital records)

- c) self-report

- d) no description

2. Was follow-up long enough for outcomes to occur

**- a) yes**

- b) no

3. Adequacy of follow-up of cohorts

**- a) complete follow-up – all subjects accounted for**

- b) subjects lost to follow-up are not described

- c) different follow-up rates between groups and no description

Overview

This table summarizes the quality assessment of the study "Evaluation of peripheral perfusion index and heart rate variability as early predictors for intradialytic hypotension in critically ill patients" using the Newcastle-Ottawa Scale.

**Prevalence and risk factors of hypotension associated with preload-dependence during intermittent hemodialysis in critically ill patients**

Selection

1. Representativeness of the exposed cohort

**- a) truly representative of the average critically ill patients undergoing renal replacement therapy in the community**

- b) somewhat representative of the average critically ill patients in the community

- c) selected group of users, e.g., nurses, volunteers

- d) no description of the derivation of the cohort

2. Selection of the non-exposed cohort

**- a) drawn from the same community as the exposed cohort**

- b) drawn from a different source

- c) no description of the derivation of the non-exposed cohort

3. Ascertainment of exposure

**- a) secure record (e.g., surgical records)**

- b) structured interview

- c) written self-report

- d) no description

4. Demonstration that outcome of interest was not present at start of study

**- a) yes**

- b) no

Comparability

1. Comparability of cohorts based on the design or analysis

**- a) study controls for the most important factor (e.g., use of norepinephrine)**

**- b) study controls for any additional factor (e.g., presence of sepsis)**

Outcome

1. Assessment of outcome

- a) independent blind assessment

**- b) record linkage (e.g., hospital records)**

- c) self-report

- d) no description

2. Was follow-up long enough for outcomes to occur

**- a) yes**

- b) no

3. Adequacy of follow-up of cohorts

**- a) complete follow-up – all subjects accounted for**

- b) subjects lost to follow-up are not described

- c) different follow-up rates between groups and no description

Overview

This table summarizes the quality assessment of the study "Prevalence and risk factors of hypotension associated with preload-dependence during intermittent hemodialysis in critically ill patients" using the Newcastle-Ottawa Scale.

**Blood volume monitoring in intermittent hemodialysis for acute renal failure**

Selection

1. Representativeness of the exposed cohort

**- a) truly representative of the average critically ill patients undergoing intermittent hemodialysis in the community**

- b) somewhat representative of the average critically ill patients in the community

- c) selected group of users, e.g., nurses, volunteers

- d) no description of the derivation of the cohort

2. Selection of the non-exposed cohort

- **a) drawn from the same community as the exposed cohort**

- b) drawn from a different source

- c) no description of the derivation of the non-exposed cohort

3. Ascertainment of exposure

**- a) secure record (e.g., surgical records)**

- b) structured interview

- c) written self-report

- d) no description

4. Demonstration that outcome of interest was not present at start of study

- **a) yes**

- b) no

Comparability

1. Comparability of cohorts on the basis of the design or analysis

- **a) study controls for the most important factor (e.g., use of norepinephrine)**

**- b) study controls for any additional factor (e.g., presence of sepsis)**

Outcome

1. **Assessment of outcome**

**- a) independent blind assessment**

**- b) record linkage (e.g., hospital records)**

- c) self-report

- d) no description

2. **Was follow-up long enough for outcomes to occur**

- **a) yes**

- b) no

3. **Adequacy of follow-up of cohorts**

**- a) complete follow-up – all subjects accounted for**

- b) subjects lost to follow-up are not described

- c) different follow-up rates between groups and no description

Overview

This table summarizes the quality assessment of the study "Blood volume monitoring in intermittent hemodialysis for acute renal failure" using the Newcastle-Ottawa Scale.

**Machine learning model to predict hypotension after starting continuous renal replacement therapy**

Selection

1. Representativeness of the exposed cohort

**- a) truly representative of the average adult patients with acute kidney injury starting CRRT in the community**

- b) somewhat representative of the average adult patients with acute kidney injury in the community

- c) selected group of users, e.g., nurses, volunteers

- d) no description of the derivation of the cohort

2. Selection of the non-exposed cohort

**- a) drawn from the same community as the exposed cohort**

- b) drawn from a different source

- c) no description of the derivation of the non-exposed cohort

3. Ascertainment of exposure

**- a) secure record (e.g., hospital records)**

- b) structured interview

- c) written self-report

- d) no description

4. Demonstration that outcome of interest was not present at start of study

**- a) yes**

- b) no

Comparability

1. Comparability of cohorts on the basis of the design or analysis

**- a) study controls for the most important factor (e.g., disease severity)**

**- b) study controls for any additional factor (e.g., comorbidities)**

Outcome

1. Assessment of outcome

**- a) independent blind assessment**

**- b) record linkage (e.g., hospital records)**

- c) self-report

- d) no description

2. Was follow-up long enough for outcomes to occur

**- a) yes**

- b) no

3. Adequacy of follow-up of cohorts

**- a) complete follow-up – all subjects accounted for**

- b) subjects lost to follow-up are not described

- c) different follow-up rates between groups and no description

Overview

This table summarizes the quality assessment of the study "Machine learning model to predict hypotension after starting continuous renal replacement therapy" using the Newcastle-Ottawa Scale.

**Predicting episodes of hypotension by continuous blood volume monitoring among critically ill patients in acute renal failure on intermittent hemodialysis**

Selection

1. Representativeness of the exposed cohort

**- a) Truly representative of critically ill patients in ICU undergoing intermittent hemodialysis (IHD)**

- b) Somewhat representative of critically ill patients in ICU

- c) Selected group of users, e.g., nurses, volunteers

- d) No description of the derivation of the cohort

2. Selection of the non-exposed cohort

**- a) Drawn from the same community as the exposed cohort**

- b) Drawn from a different source

- c) No description of the derivation of the non-exposed cohort

3. Ascertainment of exposure

**- a) Secure record (e.g., hospital records)**

- b) Structured interview

- c) Written self-report

- d) No description

4. Demonstration that outcome of interest was not present at start of study

**- a) Yes**

- b) No

Comparability

1. Comparability of cohorts on the basis of the design or analysis

**- a) Study controls for the most important factor (e.g., severity of illness)**

**- b) Study controls for any additional factor (e.g., comorbidities)**

Outcome

1. Assessment of outcome

**- a) Independent blind assessment**

**- b) Record linkage (e.g., hospital records)**

- c) Self-report

- d) No description

2. Was follow-up long enough for outcomes to occur

**- a) Yes**

- b) No

3. Adequacy of follow-up of cohorts

**- a) Complete follow-up – all subjects accounted for**

- b) Subjects lost to follow-up not described

- c) Different rates of follow-up among groups and no description

Overview

This table summarizes the quality assessment of the study "Predicting episodes of hypotension by continuous blood volume monitoring among critically ill patients in acute renal failure on intermittent hemodialysis" using the Newcastle-Ottawa Scale.

Overview

This table summarizes the quality assessment of the provided studies using the Newcastle-Ottawa Scale:

Study 1: *Prevalence and risk factors of hypotension associated with preload-dependence during intermittent hemodialysis in critically ill patients*

Selection: ★★★★

Comparability: ★★

Outcome: ★★★

Study 2: *Prediction of hemodynamic tolerance of intermittent hemodialysis in critically ill patients: a cohort study*

Selection: ★★★★

Comparability: ★★

Outcome: ★★★

Study 3: *Machine learning model to predict hypotension after starting continuous renal replacement therapy*

Selection: ★★★★

Comparability: ★★

Outcome: ★★★

Study 4: *Bedside prediction of intradialytic hemodynamic instability in critically ill patients: the SOCRATE study*

Selection: ★★★★

Comparability: ★★

Outcome: ★★★

Study 5: *Evaluation of peripheral perfusion index and heart rate variability as early predictors for intradialytic hypotension in critically ill patients*

Selection: ★★★★

Comparability: ★★

Outcome: ★★★

Study 6: *Predicting episodes of hypotension by continuous blood volume monitoring among critically ill patients in acute renal failure on intermittent hemodialysis*

Selection: ★★★★

Comparability: ★★

Outcome: ★★★

Study 7: *Blood volume monitoring in intermittent hemodialysis*

*for acute renal failure*

Selection: ★★★★

Comparability: ★★

Outcome: ★★★

*Use of online blood volume and blood temperature monitoring during haemodialysis in critically ill patients with acute kidney injury: a single-centre randomized controlled trial*

*
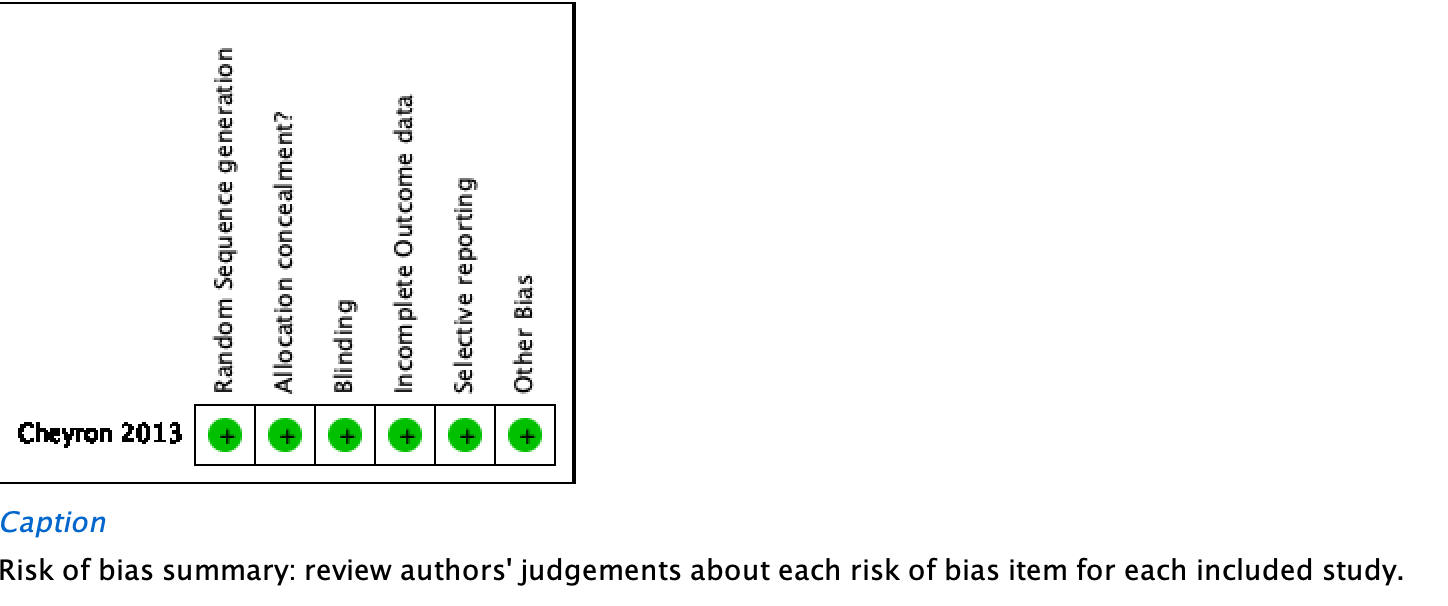
*

*
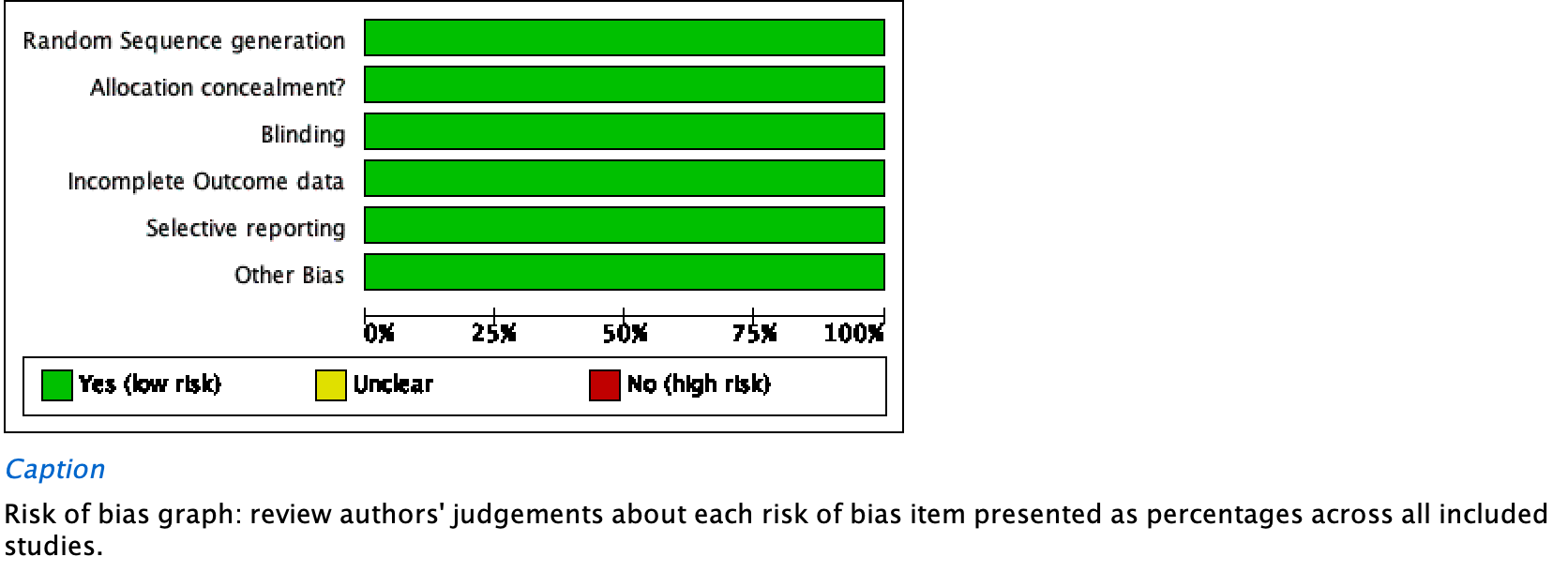
*
